# Editorial Comment: Bladder Irrigation with Tap Water to Reduce Antibiotic use for Urinary Tract Infections in Catheter Users

**DOI:** 10.1590/S1677-5538.IBJU.2025.9912

**Published:** 2025-04-15

**Authors:** Stefan den Hoedt, Felice E. E. van Veen, Jeroen R. Scheepe, Bertil F. M. Blok

**Affiliations:** 1 Department of Urology Erasmus Medical Center Rotterdam Netherlands Department of Urology, Erasmus Medical Center, Rotterdam, The Netherlands

BJU Int. 2025 Feb;135(2):286-294

DOI: 10.1111/bju.16552 | ACCESS: 39414620

## COMMENT

This prospective study included patients with an indwelling catheter or performing clean intermittent catheterization (CIC), who presented recurrent urinary tract infections (UTI) between July 2022 and March 2024, to evaluate the safety and efficacy of tap water bladder irrigation (BI) in reducing antibiotic use. Patients started daily irrigation at the onset of UTI symptoms and used a tapering schedule only in cases of uncomplicated UTI. The results were compared between a 3-month period before and after tap water BI.

Sixty patients (66.7% male, 83.3% performing CIC) were included. Antibiotic use decreased on average by 38.1% and catheter-associated UTIs by 37.9%. There was no increase in the incidence of UTIs with systemic symptoms or UTI-related hospitalizations. Furthermore, no differences were observed in health-related quality of life.

Given the significant annual incidence rate of UTI in patients that rely on bladder catheterization (1, 2), the results of the ‘proof of concept’ study place tap water BI as a promising and affordable alternative for these patients. Nevertheless, further studies are still needed not only to confirm safety and efficacy of this method with properly designed randomized studies but also to determine whether tap water from non-European countries would be feasible due to inherent differences in its composition and the need for chlorine to treat impurities (3).
